# A critical review to grading systems and recommendations of traditional Chinese medicine guidelines

**DOI:** 10.1186/s12955-020-01432-x

**Published:** 2020-06-09

**Authors:** Juan Li, Bin Li, Xin-ke Zhao, Jia-yin Tu, Yingdong Li

**Affiliations:** 1 School of Basic Medical Sciences of Lanzhou University, Lanzhou, 730000 China; 2 The 940th Hospital of Joint Logistics Support Force of People’s Liberation Army, Lanzhou, China; 3 School of Traditional Chinese and Western Medicine of Gansu University of Chinese Medicine, Lanzhou, 730000 China; 4 The Clinic of Air Force Base, Lanzhou Military, 730000 China

**Keywords:** Traditional Chinese medicine, TCM, Clinical practice guidelines, Grading systems

## Abstract

**Objectives:**

To investigate how many traditional Chinese medicine (TCM) guidelines adopted a grading system and the differences among them, and the distribution of level of evidence used to support TCM recommendations.

**Methods:**

A comprehensive search of relevant guideline webpages and literature databases were undertaken from inception to August 2018 to identify guidelines including TCM interventions. Two independent reviewers extracted the information about grading systems and recommendations.

**Results:**

One hundred forty-two TCM guidelines were included, among which, 68 (47.9%) adopted a total of eight grading systems. The definitions, letters, and codes among these systems varied significantly. A total of 1284 recommendations were extracted from included TCM guidelines. More than 60% recommendations were based on a low and very low level of evidence (level C:33.4% and level D: 30.2%). Only 7.8% recommendations were rated as strong recommendation (grade I), while 76.2% recommendations were rated as conditional recommendation (grade II).

**Conclusions:**

Various grading systems were used in TCM guidelines, which might confuse guideline users. The low proportion of high level of evidence in TCM recommendations might downgrade the confidence to TCM interventions.

## Introduction

China is the only country where western medicine and traditional Chinese medicine (TCM) are practiced alongside each other at every level of the healthcare system. and traditional Chinese treatments account for about 40% of the total [1]. Over the past two decades, an increasing number of TCM guidelines have been developed by academic associations and government organizations in China [2, 3]. The Chinese guideline clearinghouse (CGC) indexed more than 1 hundred TCM guidelines. At least a thousand recommendations were generated by providing enormous diagnostic and therapeutic options for clinical practitioners.

When guideline panels, especially evidence-based guideline panels decide to recommend an interventional or diagnostic option for guidelines users, they must make judgments about the quality of evidence and strength of recommendations based on the evidence related to the specific context and other factors. The quality of evidence reflects the extent to which confidence in an estimate of the effect is adequate to support a particular recommendation [4, 5]. The strength of a recommendation indicates the extent to which one can be confident that adherence to the recommendation will do better than harm [4]. Normally, the higher the quality of evidence, the more likely a strong recommendation can be made [6]. However, several previous surveys indicated that many recommendations in important domains (such as oncology, cardiology, infectious disease, and screening) were based on a low level of evidence [7–9], even in the World Health Organization (WHO) guidelines, strong recommendations based on low levels of evidence are frequent [10].

The quality of evidence and strength of recommendations are necessary as they could help to communicate a clear message, so as to help guideline users, readers and stakeholders to understand recommendations quickly and concisely, and more and more international guidelines organizations, such as Scottish Intercollegiate Guidelines Network (SIGN), National comprehensive cancer network (NCCN), National Institute for Health and Care Excellence (NICE) and WHO are applying a grading system to rate the quality of evidence and strength to their recommendations. However, the grading systems adopted by different guideline organizations varied in the definitions, letters, and codes [11], for example, the codes of GRADE system [12], Oxford system [13], SIGN system [14], and NCCN system [15], fall primarily into three categories: letters (e.g., A, B, C, etc.), numbers (e.g., I, II, III, etc.) and mixed letters and numbers (e.g., Ia, Ib, IIa, etc.).

Limited studies have focused on the varied grading systems in guidelines. Holger J, et al. [16] investigated the letters, numbers, symbols and words of grading systems from different guideline organizations in English-speaking countries. Andre I, et al. [17] investigated the level of evidence of recommendations in pediatrics guidelines. While no published study was found to investigate grading systems in TCM guidelines. Thus, this study aimed to investigate: 1) how many TCM guidelines adopted a grading system and the differences among them; 2) the distribution of level of evidence and strength of recommendation in TCM guidelines.

## Methods

### Identification of guidelines

Chinese Guideline Clearinghouse and PubMed were searched as an international database, and Wanfang Data Knowledge Service Platform, VIP Online Publishing Platform, China National Knowledge Infrastructure (CNKI) and SinoMed were searched as domestic databases for TCM guideline. Search terms in electronic databases included “guidelines”, “statement”, “recommendations”, “traditional medicine”, “traditional Chinese medicine”, and “Chinese herbal medicine”. Additional file 1 showed the detailed searching strategy. The searches were initially conducted in December 2016 and updated in August 2018 to include more TCM guidelines. Other sources such as Google, Amazon and Dang-dang website were searched for TCM guidelines published in books. Additionally, the reference lists of obtained guidelines were searched to include more potential guidelines.

### Selection of guidelines

The including criteria were as follows: (1) complete guideline text is available in English or Chinese, and (2) guideline contains recommendations regarding TCM interventions. If a guideline had updates, only the most recent version would be assessed. The following literature was excluded: duplicate guidelines, guidelines for patients, editorials, translations of guidelines, secondary or multiple publications and short summaries. We also excluded “de novo” or adaptations of external guidelines, as they did not generate their own recommendations.

### Data extraction

For each included TCM guideline, two reviewers will independently extract the following information by using a standard form: 1) characteristic information: guideline organizations (i.e. ministry of health, medical doctor association, Chinese medical institute, China Academy of TCM and hospital), year of publication, journal, publication type (e.g. CSCD journal, non-CSCD journal, and book), scope of guideline (prevention and treatment, prevention, diagnosis and treatment, treatment, technology and comprehensive) and funding information, classified into four categories: industry, government, academic association and not reported; 2) grading systems information including: any form of grading systems used for rating the level of evidence and/or strength of recommendations, and the codes, letters, numbers, and symbols of the grading system were also extracted when available; 3) recommendation information: the exact recommendations of each TCM guideline were extracted and standardized into treatment or diagnostic categories. The Consensus was reached by discussing when recommendations were not obvious in guidelines, and disagreements were settled by discussing with a third author (Y.D.L).

### Primary measures

The proportion of different levels of evidence and strength of recommendations in TCM guidelines was estimated and compared among subgroups. Considering various grading systems might be used to evaluate the level of evidence and strength of recommendations, a composite grading system was generated to represent all recommendations according to a study published in *Chest* [18].

The level of evidence was classified into four categories: grade A (high), the evidence used for supporting the recommendation was based on randomized controlled trials without important limitations, or meta-analysis; grade B (moderate), evidence behind the recommendation was based on randomized controlled trials with important limitations, or upgraded observational studies; grade C (low), evidence for recommendation was based on non-randomized studies, cohort or case-control studies, case series; and grade D (very low), the recommendation was made by expert opinion.

The strength of recommendation was adapted into three categories: level I (strong recommendation), recommendation can apply to most patients in many circumstances; level II (conditional recommendation), the best action may differ depending on circumstances or patients’ or societal values; and level UG, insufficient evidence on which to formulate a recommendation.

### Subgroup analysis

The following subgroup analyses were conducted to compare the difference of level of evidence and strength of recommendations in TCM guidelines including: 1) year of publication (e.g. 2003–2007 vs. 2008–2012 vs. 2013–2016 vs. 2017–2018); 2) type of recommendation (treatment vs. diagnosis); 3) form of publication (CSCD indexed journal vs. non-CSCD indexed journal vs. book); and source of funding (any funding vs. not reporting).

### Data analysis

Statistic tests of significance might not be necessary as we included the entire cohort of TCM guidelines. Instead, we performed a descriptive analysis using the calculation of the percent distributions of recommendations among quality of evidence and strength of recommendations. Agreement between each reviewer’s data extraction was tested by using a two-way ANOVA with single-rater two-way intra-class correlation coefficients (ICCs). According to Landis and Koch [19], the degree of agreement between 0.01 and 0.20 was deemed minor, 0.21–0.40 fair, 0.41–0.60 moderate, 0.61–0.80 substantial, and 0.81–1.00 very good. A value of *P* < 0.05 denoted statistical significance. All tests were two-sided. Statistical analyses were conducted using SPSS version 19.0 (SPSS Inc., Chicago, IL, USA).

## Results

### Search results

**Fig. 1:**
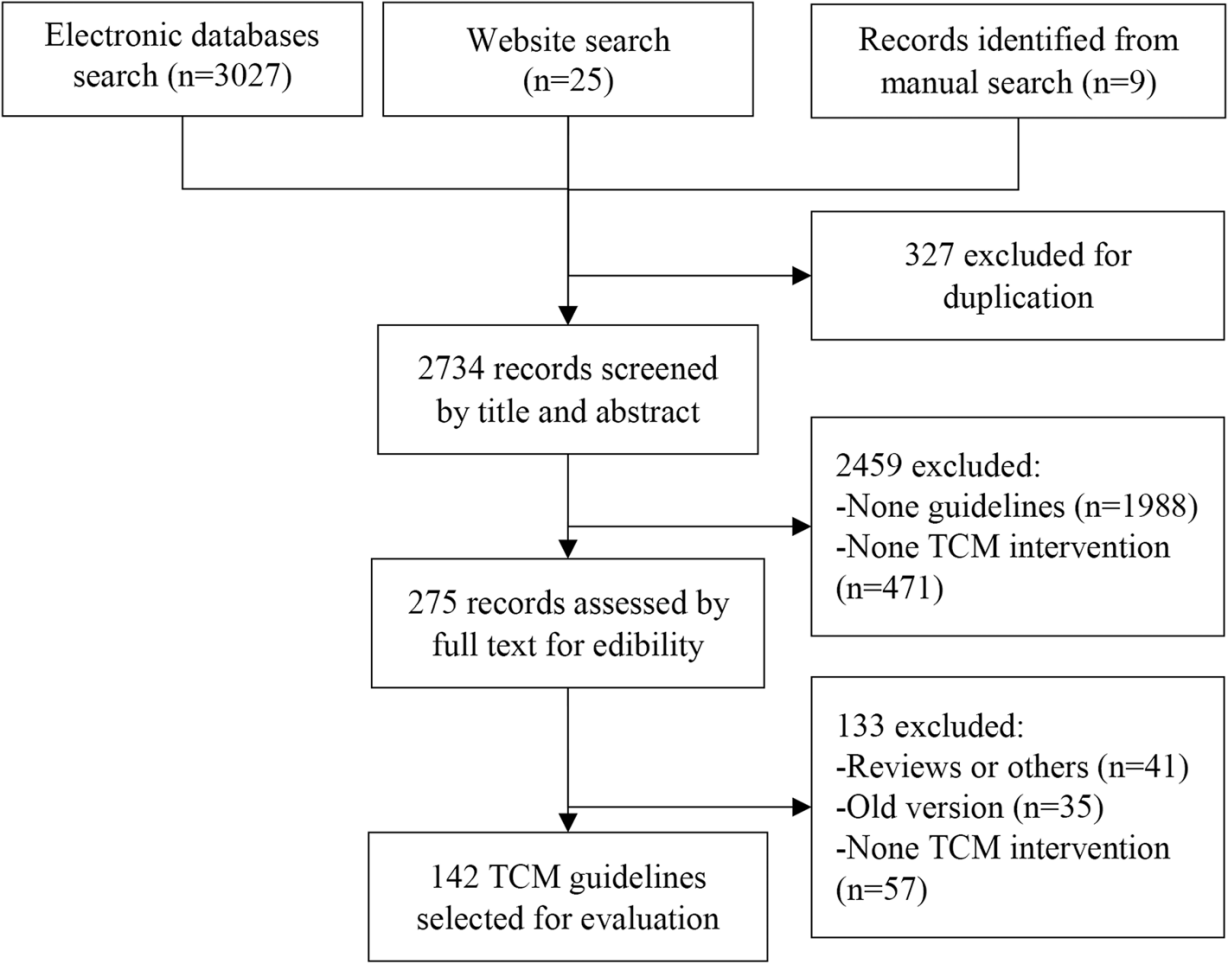
Flow chart of selecting TCM guidelines

We yielded 2380 records from databases, guideline websites and manual searches after excluding duplicated records, of which 166 records were considered to be potentially relevant; after selection, a total of 142 TCM guidelines were satisfied the inclusion criteria (Fig. 1).

### Guideline characteristics

**Fig. 2:**
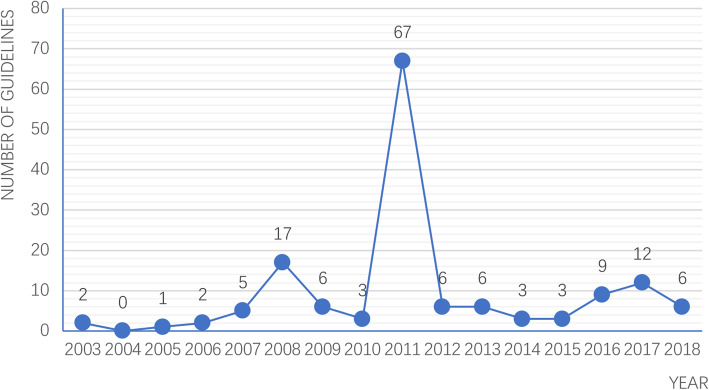
TCM guidelines published in different years

Table 1 showed the characteristics of 142 TCM guidelines. 69.8% (*n* = 99) TCM guidelines published in 2008–2012 (Fig. 2), 50.0% (*n* = 71) TCM guidelines developed by Chinese medical institute and 197% (*n* = 28) developed by China Academy of TCM. 86.6% (*n* = 123) TCM guidelines focused on diagnosis and treatment. In terms of funding, 53.5% (*n* = 76) TCM guidelines were supported by the government, and 19.7% (*n* = 28) funded by medical association, however, none TCM guidelines reported the conflicts of interest statement. The ICC value for data extraction process was 0.87, 95% confidence interval 0.83 to 0.90, indicating a good agreement between reviewers.

**Table 1:** The characteristics of included studies

Categories	No of guidelines (%)
Scope of guidelines	
-Prevention and treatment	7 (5.0)
-Prevention	1 (0.7)
-Diagnosis and treatment	123 (86.6)
-Treatment	5 (3.5)
-Technology	5 (3.5)
-Comprehensive^a^	1 (0.7)
Development organization	
-Ministry of health	3 (2.1)
-Medical doctor association	4 (2.8)
-Chinese Medical institute	71 (50.0)
-China Academy of TCM	28 (19.7)
-Hospital	34 (23.9)
-Comprehensive^b^	2 (1.5)
Publication	
-CSCD Journal	26 (18.3)
-Non CSCD journal	88 (62.0)
-Book	28 (19.7)
Funding	
-Industry	0
-Government	76 (53.5)
-Academic association	28 (19.7)
-Not reported	38 (26.8)

^a^Including two or more scopes in a guideline; ^b^ Developed by two or more organizations in a guideline

### Evidence grading systems

In the 142 TCM guidelines, 52.1% did not adopt any grading system, eight grading systems were found in the rest 47.9% TCM guidelines. Table 2 showed the codes of quality of evidence and strength of recommendation for reported grading systems. The most often used system was TCM grading system developed by Liu Jianping (Director of evidence-based center of Beijing University of Chinese medicine) team [20], which was used in 53 TCM guidelines, followed by GRADE system [12] used in four TCM guidelines. The other grading systems such as SIGN system [14], Agency for Healthcare Research and Quality (AHRQ) system [21], Oxford system [13], North of England Evidence Based Guidelines Development Project (NEEBGDP) system [22], and David Sackett system [23] were used by only one TCM guideline respectively.

**Table 2:** The codes of different grading systems in TCM guidelines

Grading systems	Codes of evidence and recommendation		Number of Guidelines (%)
	Quality of evidence	Strength of recommendation	
TCM grading system	Ia, Ib, IIa, IIb, IIIa, IVb, IV, V	A, B, C	53 (77.9)
GRADE system	A, B, C, D	1, 2	4 (5.9)
Oxford system	1a, 1b, 1c, 2a, 2b, 2c, 3a, 3b, 4, 5	A, B, C, D	4 (5.9)
Adapted David Sackett system	A, B, C	I, IIa, IIb, III, IV	3 (4.3)
SIGN system	1, 2, 3, 4	A, B, C, D, E	1 (1.5)
Adapted NEEBGDP system	I, II, III, IV	A, B, C, experts opinion	1 (1.5)
Adapted AHRQ system	Ia, Ib, II, III, IVa, IVb	A+, A-, B+, B-, C, D	1 (1.5)
AHRQ system	Ia, Ib, IIa, IIb, III, IV	A, B, C, D	1 (1.5)

### Level of evidence and strength of recommendation

A total of 1284 recommendations were extracted from 142 included guidelines. Of recommendations with levels of evidence, more than 60% based on level C and D evidence (33.4 and 30.2%, respectively) (Table 3). Among recommendations with a strength of recommendation, only 7.8% were rated as strong (level I), 76.2% as weak (level II), and 16.0% as level UG.

**Table 3:** Distribution of the Strength of Recommendation and Level of Evidence among TCM Guidelines

Subgroups	No of recommendations	Strength of Recommendation, No. (%)			Level of Evidence, No. (%)			
		I	II	UG	A	B	C	D
Year of publication								
2003–2007	82	11 (13.4)	45 (54.9)	26 (31.7)	9 (11.0)	30 (36.6)	21 (25.6)	22 (26.8)
2008–2012	984	68 (6.9)	776 (78.9)	140 (14.2)	101 (10.3)	228 (23.2)	341 (34.7)	314 (31.9)
2013–2016	100	5 (5.0)	78 (78.0)	17 (17.0)	11 (11.0)	25 (25.0)	41 (41.0)	23 (23.0)
2017–2018	118	16 (13.6)	79 (66.9)	23 (19.5)	21 (17.8)	42 (35.6)	26 (22.0)	29 (24.6)
Total	1284	100 (7.8)	978 (76.2)	206 (16.0)	142 (11.1)	325 (25.3)	429 (33.4)	388 (30.2)
Type of recommendation								
Treatment	905	73 (8.0)	678 (75.0)	154 (17.0)	97 (10.7)	224 (24.8)	267 (29.5)	317 (35.0)
Diagnosis	389	28 (7.2)	309 (79.4)	52 (13.4)	45 (11.6)	101 (26.0)	162 (41.6)	81 (20.8)
Total	1284	100 (7.8)	978 (76.2)	206 (16.0)	142 (11.1)	325 (25.3)	429 (33.4)	388 (30.2)
Type of publication								
CSCD indexed journal	364	28 (7.7)	280 (77.1)	55 (15.2)	43 (11.8)	85 (23.1)	117 (32.2)	119 (32.8)
Non-CSCD indexed journal	680	62 (9.1)	511 (75.1)	107 (15.7)	76 (11.2)	175 (25.7)	221 (32.5)	208 (30.6)
Book	250	10 (4.0)	196 (78.4)	44 (17.6)	23 (9.2)	66 (26.4)	91 (36.4)	70 (28.0)
Total	1284	100 (7.8)	978 (76.2)	206 (16.0)	142 (11.1)	325 (25.3)	429 (33.4)	388 (30.2)
Source of funding								
Any funding	924	74 (7.9)	675 (73.1)	175 (19.0)	105 (11.4)	257 (27.8)	334 (36.2)	228 (24.6)
Not reported	370	27 (7.3)	312 (84.3)	31 (8.4)	37 (10.0)	68 (18.4)	95 (25.7)	170 (45.9)
Total	1284	100 (7.8)	978 (76.2)	206 (16.0)	142 (11.1)	325 (25.3)	429 (33.4)	388 (30.2)

Subgroup analyses of year of publication, types of recommendation, form of publication, and source of funding showed no significant difference in strong recommendation (grade I) and high level of evidence (grade A), instead, recommendations from any funding got a higher proportion in grade B and C level of evidence (27.8% vs 18.4%; 36.2% vs 25.7% respectively) and lower proportion in grade D level of evidence (24.6% vs 45.9%).

## Discussion

Our results showed that eight evidence grading systems were used in 47.8% TCM guidelines, while the rest 52.1% did not sue any evidence grading system. For the eight evidence grading systems, the different codes, letters, and level of evidence are likely to lead to confuses and even misunderstanding for guideline users when using TCM guidelines.

The definitions to the eight grading systems also varied a lot, for example, the GRADE system graded the quality of evidence according to bias which might decrease the confidence of included studies, that is to say, even RCTs might be at low quality of evidence if they were at high risk of bias [12]. On the other hand, the other systems such as NEEBGDP and Oxford system, they classified the level of evidence according to study design, which led to that RCTs would always be graded as high quality of evidence whether they were at high or low risk of bias. Similar situations happened in the strength of recommendations, it did not classify expert opinions as a level of evidence in GRADE system, but it was a level in the TCM grading system and NEEBGDP system.

Besides, the codes from the eight grading systems were very different (Table 1), for example, the “A, B, C, D” codes were used to reflect the quality of evidence in the GRADE system, and the adapted David Sackett system, but they were also used to indicate the strength of recommendation in the TCM grading system, SIGN system, adapted NEEBGDP system, and AHRQ system. We could imply that fresh health care professionals, especially medical students, perhaps, are easily puzzled by the information of different grading systems conveyed. Thus, various grading systems may not fulfill their original intention. Indeed, if the same code, used by different systems, represents different meanings, may result in bewilderment and incomprehension.

Considering different grading systems would generate different results of level of evidence and strength of recommendation, which could contribute to challenges of implementing of TCM guidelines, therefore a standardized and well-recognized grading system is needed for TCM guidelines. A study evaluated the effect of presenting a recommendation in a clinical practice guideline using different grading systems to determine to what extent the system used changes the clinician’s eventual response to a particular clinical question, they found the clinician’s decision to use a therapy was influenced most by the GRADE system [24]. It would be valuable to conduct a similar study in TCM guidelines to determine which grading system influence most to TCM clinicians.

Some international guideline organizations such as SIGN and AHRQ had realized the importance of reaching consensus to standardized grading system and began to adopt the GRADE system in their new guidelines. The GRADE system was developed by GRADE working group in 2000, which has been adopted by more than 100 national and international organizations [25]. Similarly, the Cochrane Collaboration now requires authors to use GRADE for all important outcomes in their systematic reviews. Hence, that might be an option for TCM guidelines to use or adapt GARDE system in the future. In addition, some Chinese researchers argued external grading systems such as GARDE and oxford might not be suitable for TCM guidelines when considering the big gaps between TCM and western medicine, for example, the construction of TCM guidelines follow some principles from ancient TCM books, some TCM treatments are only used in Asian counties, hence, they generated a new grading system for TCM guideline, i.e., the TCM grading system reported in this study. Now the TCM grading system was updated in 2019 to make it more approachable and understandable for users [26].

Another important issue is that we found more than 50% of TCM guidelines did not adopt any grading system. This could limit the application of TCM guidelines in practice. It is almost a consensus among international guideline organizations that a unique systematic approach to grade evidence and strength of recommendations can minimize bias, aid interpretation and promote the implementation of guidelines [4], as grading systems could promote guidelines delivering a clear message so as to help guideline readers understand recommendations quickly and concisely. According to a survey, there are more than 60 grading systems with extremely wide variations to quality of evidence and strength of recommendations [27], so it still has a long way to reach a consensus of using a standard system in different guideline organizations. Most TCM guideline developers didn’t seem to realize the advantages of using grading systems. Good news is that one guideline handbook for integrative medicine was published in 2016 [28] and using the GRADE system to rate the quality of evidence and strength of recommendations was encouraged, which will promote the use of grading systems in the coming TCM guidelines.

Normally, the higher the quality of evidence, the more likely a strong recommendation can be made [4]. However, in this study, only 7.8% recommendations, based on a high level of evidence (grade I, meta-analysis and RCT), which would downgrade our confidence to the TCM interventions in some degree. According to Deng et al’s study [29], the low proportion of meta-analysis and RCT referred by TCM recommendations was associated with insufficient search of electronic databases, and poor rigor of development process. We should also notice with the quality of meta-analysis and RCTs referred by TCM guidelines, Yao et al. [30] study showed that the quality of TCM meta-analyses published in 2010–2012 was significantly lower than non-TCM meta-analyses. And Wu et al. [31] found that more than 90% of RCTs published in core Chinese journals lacked an adequate description of randomization. They found most trials, despite being claimed to be RCTs, did not fulfill the criteria of a real RCT, which suggested a lack of adequate understanding of rigorous clinical trial design and conducting among the investigators.

The conflicts of interest in guidelines, which defines as any interest held by an expert that may affect or reasonably be perceived to affect the expert’s objectivity and independence in providing advice, can influence the level of evidence and strength of recommendations [32]. WHO divides the conflicts of interest into three categories: financial, academic and public positions [33]. A financial relationship could impact an individual’s ability to approach a scientific question with an open mind, and academic activities could create the potential for an attachment to a specific point of view that could unduly affect an individual’s judgment about a specific recommendation [33]. Although 73.2% of TCM guidelines reported the funding source, none of them reported the conflicts of interest. We might exclude the possibility of financial conflicts of interest in TCM guidelines as none of them reported funding from pharmaceutical companies, however academic and public conflicts of interest remain unclear. More importantly, there are still 26.8% TCM guidelines did not report any information about funding or conflicts of interest, the insufficient transparency to funding and conflicts of interest statement would downgrade the confidence to TCM interventions.

Subgroup analyses indicated that the proportion of the high level of evidence from TCM guidelines published in CSCD journals was not better than in non-CSCD journals. This might indicate that the peer review process in CSCD journals is probably not more rigorous than in non-CSCD journals. This is surprising because CSCD journals are required to meet more criteria, compared to those in non-CSCD journals.

Addressing the limitations of existing TCM guidelines, a standardized grading system developed by multidisciplinary experts is needed. Using the same grading system and codes will make TCM guidelines more understandable. Moreover, informing TCM recommendations underlying the evidence-based approach, as well as using systematic reviews to support recommendations will make TCM guidelines more trustworthy.

## Strengths and limitations

Our overall findings have some strengths. Firstly, this study is the first to examine the evidence type and grading systems of recommendations in TCM guidelines. Secondary, each grading system has been appropriately compared according to their codes and definitions, which provided clear information about the differences among them. Thirdly, and subgroups analyses were conducted to detect the variation of different groups among level of evidence and strength of recommendation. Nonetheless, our study has several limitations. We included TCM guidelines in journals and books, but we may have missed guidelines published in other forms such as web page, which might understate the performance of TCM guidelines. Second, our study could not establish the causality between the poor performance and the characteristics of TCM guidelines. Besides, the evidence level of each recommendation was appropriately re-classified according to evidence type by evaluators, which might lead to some bias to interpret the quality of TCM guidelines.

## Conclusions

Various grading systems were used in TCM guidelines, which might confuse guideline users. The low proportion of high level of evidence in recommendations could downgrade the confidence to TCM interventions. A standardized grading system should be established in TCM guidelines. And more high level of evidence should be used to enhance the confidence of recommendations and promote the dissemination and implementation of TCM guidelines.

## Supplementary information

**Additional file 1.** Search strategies for TCM guidelines.

**Additional file 2.** The characteristic information of included TCM guidelines.

## Data Availability

All data analyzed during this study are included in this published article’s Additional file 1.

## Abbreviations

TCM: Traditional Chinese Medicine
AHRQ: Agency for Healthcare Research and Quality
NEEBGDP: North of England Evidence Based Guidelines Development Project
WHO: World Health Organization
CGC: Chinese guideline clearinghouse
CNKI: China National Knowledge Infrastructure
RCT: Randomized controlled trial
CCT: Controlled clinical trial
CSCD: Chinese Science Citation Database
GRADE: Grading of Recommendations Assessment, Development and Evaluation
SIGN: Scottish Intercollegiate Guidelines Network
NCCN: National Comprehensive Cancer Network

## References

[1] Hesketh T, Zhu WX (1997). Health in China. Traditional Chinese medicine: one country, two systems. BMJ.

[2] Chen YL, Yao L, Xiao XJ (2012). Quality assessment of clinical guidelines in China: 1993–2010. Chin Med J.

[3] Chen YL, WANG C, Shang H, Yang K, Susan L (2018). Clinical practice guidelines in China. BMJ.

[4] Atkins D, Best D, Briss PA, Eccles M, Falck Y (2004). Grading quality of evidence and strength of recommendations. BMJ.

[5] Guyatt GH, Oxman AD, Vist GE, Kunz R, Falck-Ytter Y (2008). GRADE: an emerging consensus on rating quality of evidence and strength of recommendations. BMJ.

[6] Andrews JC, Schünemann HJ, Oxman AD, Pottie K, Meerpohl JJ (2013). GRADE guidelines: 15. Going from evidence to recommendation-determinants of a recommendation's direction and strength. J Clin Epidemiol.

[7] Tricoci P, Allen JM, Kramer JM, Califf RM, Smith SC (2009). Scientific evidence underlying the ACC/AHA clinical practice guidelines. JAMA.

[8] Poonacha TK, Go RS (2011). Level of scientific evidence underlying recommendations arising from the National Comprehensive Cancer Network clinical practice guidelines. J Clin Oncol.

[9] Lee DH, Vielemeyer O (2011). Analysis of overall level of evidence behind Infectious Diseases Society of America practice guidelines. Arch Intern Med.

[10] Alexander PE, Brito JP, Neumann I, Gionfriddo MR, Bero L (2016). World Health Organization strong recommendations based on low-quality evidence (study quality) are frequent and often inconsistent with GRADE guidance. J Clin Epidemiol.

[11] Chen YL, Li YP, Du L, Wang L, Wen J (2008). Evolution of levels of evidence and strength of recommendations in medical research. Chin Evid Based Med.

[12] Schunemann H, Brozek J, Oxman A (2013). GRADE handbook for grading quality of evidence and strength of recommendation.

[13] Oxford Centre for Evidence-Based Medicine Levels of Evidence Working Group. The Oxford 2011 levels of evidence. Available at: www.cebm.net/index.aspx?o=5653. Accessed at 25 July 2018.

[14] Scottish Intercollegiate Guidelines Network (SIGN).http://www.sign.ac.uk/. Accessed at 25 July 2018.

[15] National comprehensive cancer network. https://www.nccn.org/professionals/physician_gls/f_guidelines.asp. Accessed at 25 July 2018.

[16] Schünemann HJ, Best D, Vist G, Oxman AD, GRADE Working Group (2003). Letters, numbers, symbols and words: how to communicate grades of evidence and recommendations. CMAJ.

[17] Isaac A, Saginur M, Hartling L, Robinson JL (2013). Quality of reporting and evidence in American Academy of Pediatrics guidelines. Pediatrics.

[18] Jiang M, Guan WJ, Fang ZF (2016). A Critical Review of the Quality of Cough Clinical Practice Guidelines. Chest.

[19] Landis RJ, Koch GG (1977). The measurement of observera agreement for categorical data. Biometrics.

[20] Liu JP (2007). The composition of evidence body of traditional Chinses medicine and recommendations for its evidence grading. Chin J Integr Med.

[21] Agency for Healthcare Research and Quality (AHRQ). http://www.ahrq.gov/. Accessed at 1 Aug 2018.

[22] Eccles M, Clapp Z, Grimshaw J, Adams PC, Higgins B (1996). Russell I: north of England evidence based guidelines development project: methods of guideline development. BMJ.

[23] Sackett DL (1986). Rules of evidence and clinical recommendations on the use of antithrombotic agents. Chest.

[24] García CAC, Pacheco Alvarado KP, Gaxiola GP (2011). Grading recommendations in clinical practice guidelines: randomised experimental evaluation of four different systems. Arch Dis Child.

[25] GRADE working group. http://www.gradeworkinggroup.org/. Accessed at 5 Aug 2018.

[26] Chen W, Fang SN, Liu JP (2019). Recommendations for clinical evidence grading on traditional Chinese medicine based on evidence body. CJITWM.

[27] West S, King V, Carey TS, et al. Systems to rate the strength of scientific evidence. Evid Rep Technol Assess (Summ). 2002;(47):1–11. PMC4781591 11979732

[28] Lu C, Yang K (2016). Guideline handbook for integrative medicine.

[29] Deng W, Li L, Wang Z, Chang X, Li R (2016). Using AGREE II to evaluate the quality of traditional medicine clinical practice guidelines in China. J Evid Based Med.

[30] Yao L, Sun R, Chen YL, Wang Q, Wei D (2016). The quality of evidence in Chinese meta-analyses needs to be improved. J Clin Epidemiol.

[31] Wu T, Li Y, Bian Z, Liu G, Moher D (2009). Randomized trials published in some Chinese journals: how many are randomized. Trials.

[32] Institute of Medicine (US). Committee on Standards for Developing Trustworthy Clinical Practice Guidelines, Graham R, Mancher M. Clinical practice guidelines we can trust[M]. Washington, DC: National Academies Press; 2011. 24983061

[33] World Health Organization. WHO handbook for guideline development[M]. World Health Organization: WHO press; 2014.

