# Supplementary material for: A critical review to grading systems and recommendations of traditional Chinese medicine guidelines
Source: Health Qual Life Outcomes. 2020 Jun 9;18:174. doi: 10.1186/s12955-020-01432-x (PMC7285562; doi:10.1186/s12955-020-01432-x)
Supplement: Supplementary file 1 — Additional file 1. Search strategies for TCM guidelines. [file 12955_2020_1432_MOESM1_ESM.docx]

**Additional file 1. Search strategies for TCM guidelines**

1. **Chinese Guideline Clearinghouse**

#1 Traditional Chinese medicine 118

1. **PubMed**

#1 "Medicine, Chinese Traditional"[Mesh] 14727

#2 “Traditional Chinese medicine” [Title/Abstract] 11029

#3 “Chinese herbal medicine” [Title/Abstract] 2382

#4 TCM [Title/Abstract] 6396

#5 “traditional medicine”Title/Abstract] 6972

#6 OR/#1-5 32000

#7 "Guideline" [Publication Type] 28195

#8 recommendation*[Title/Abstract] 174326

#9 statement[Title/Abstract] 30175

#10 guideline*[Title/Abstract] 228710

#11 OR/#7-10 403229

#12 #6 and #11 493

1. **Wanfang Data Knowledge Service Platform**

#1 指南[主题] 58939

#2 指引[主题] 34903

#3 共识[主题] 67456

#4 OR/#1-3 143748

#5 中医药[主题] 114089

#6 #4 and #5 790

1. **VIP Online Publishing Platform**

#1 指南[题名或关键词] 50816

#2 指引[题名或关键词] 6073

#3 共识[题名或关键词]13148

#4 OR/#1-3 73612

#5 中医药[题名或关键词] 216590

#6 #4 and #5 272

1. **China National Knowledge Infrastructure**

#1 指南[主题] 28348

#2 指引[主题] 4629

#3 共识[主题] 8905

#4 OR/#1-3 40016

#5 中医药[主题] 107954

#6 #4 and #5 538

1. **SinoMed**

#1 "指南"[不加权:扩展] 672

#2 "指引"[常用字段:智能] 2696

#3 "共识"[常用字段:智能] 10096

#4 "指南"[常用字段:智能] 26567

#5 OR/#1-4 37862

#6 中医药[不加权:扩展] 120643

#7 #5 and #6 143

1. **Google**

“Traditional Chinese medicine” and Guidelines (the first 200 records were screened)

1. **Amazon**

“Guidelines” were searched as key word for TCM guidelines published as books

1. **Dangdang**

“Guidelines” were searched as key word for TCM guidelines published as books
