# Supplementary material for: A critical review to grading systems and recommendations of traditional Chinese medicine guidelines
Source: Health Qual Life Outcomes. 2020 Jun 9;18:174. doi: 10.1186/s12955-020-01432-x (PMC7285562; doi:10.1186/s12955-020-01432-x)
Supplement: Supplementary file 2 — Additional file 2. The characteristic information of included TCM guidelines. [file 12955_2020_1432_MOESM2_ESM.docx]

Additional file 2. The characteristic information of included TCM guidelines

| 序号 | 指南题目 | 发表杂志 | 发表年份 | 更新 | 制定者 | 分类 | 检索 | 检索词 | 参考文献数量 | 利益冲突 | 资助 |
| --- | --- | --- | --- | --- | --- | --- | --- | --- | --- | --- | --- |
| 1 | 中医儿科临床诊疗指南·小儿口疮 | 中医儿科杂志 | 2018 | 已更新 | 医院 | 诊疗 | 是 | 是 | 17 | 无 | 国家中医药管理局《2014年中医药部门公共卫生服务补助资金中医药标准制修订项目 (051) |
| 2 | 中医儿科临床诊疗指南·小儿胃炎(修订) | 中医儿科杂志 | 2018 | 已更新 | 医院 | 诊疗 | 是 | 是 | 21 | 无 | 国家中医药管理局《2014年中医药部门公共卫生服务补助资金中医药标准制修订项目 (250) |
| 3 | 冠心病心绞痛介入前后中医诊疗指南 | 中国实验方剂学杂志 | 2018 | 未提及 | 学会 | 诊疗 | 否 | 否 | 11 | 未提及 | 国家重点基础研究发展计划 (973计划) 项目 (No.2003CB517103) 和国家自然科学基金重大研究计划 (No.90709048) |
| 4 | 中医儿科临床诊疗指南·胎黄(修订) | 中医儿科杂志 | 2018 | 已更新 | 医院 | 诊疗 | 是 | 是 | 23 | 无 | 国家中医药管理局《2014年中医药部门公共卫生服务补助资金中医药标准制修订项目 (88) |
| 5 | 中医儿科临床诊疗指南·蛲虫病(修订) | 中医儿科杂志 | 2018 | 已更新 | 医院 | 诊疗 | 是 | 是 | 9 | 无 | 国家中医药管理局《2014年中医药部门公共卫生服务补助资金中医药标准制修订项目 (64) |
| 6 | 中医儿科临床诊疗指南·小儿遗尿症(修订) | 中医儿科杂志 | 2018 | 已更新 | 医院 | 诊疗 | 是 | 是 | 19 | 无 | 国家中医药管理局《2014年中医药部门公共卫生服务补助资金中医药标准制修订项目 (05) |
| 7 | 中医儿科临床诊疗指南·小儿癫痫(修订) | 中医儿科杂志 | 2017 | 已更新 | 医院 | 诊疗 | 是 | 是 | 16 | 无 | 国家中医药管理局《2014年中医药部门公共卫生服务补助资金中医药标准制修订项目 (12) |
| 8 | 中医儿科临床诊疗指南·蛔虫病(修订) | 中医儿科杂志 | 2017 | 已更新 | 医院 | 诊疗 | 是 | 是 | 14 | 无 | 国家中医药管理局《2014年中医药部门公共卫生服务补助资金中医药标准制修订项目 (63) |
| 9 | 甲状腺手术针药复合麻醉应用指南 | 世界中医药 | 2017 | 未提及 | 医院 | 技术 | 否 | 否 | 30 | 未提及 | 国家重点基础研究发展计划 (973计划) 项目 (2013CB531904) |
| 10 | .中医儿科临床诊疗指南·儿童多动症(修订) | 中医儿科杂志 | 2017 | 已更新 | 医院 | 诊疗 | 是 | 是 | 32 | 无 | 国家中医药管理局《2014年中医药部门公共卫生服务补助资金中医药标准制修订项目 (60) |
| 11 | 中医儿科临床诊疗指南·细菌性痢疾(制订) | 中医儿科杂志 | 2017 | 否 | 医院 | 诊疗 | 是 | 是 | 23 | 无 | 国家中医药管理局《2014年中医药部门公共卫生服务补助资金中医药标准制修订项目 (145) |
| 12 | 中医治未病实践指南·药膳干预小儿脾虚质(制订) | 中医儿科杂志 | 2017 | 否 | 医院 | 诊疗 | 是 | 是 | 26 | 无 | 国家中医药管理局《2014年中医药部门公共卫生服务补助资金中医药标准制修订项目 (44) |
| 13 | 中医儿科临床诊疗指南·疳证(修订) | 中医儿科杂志 | 2017 | 已更新 | 医院 | 诊疗 | 是 | 是 | 14 | 无 | 国家中医药管理局《2014年中医药部门公共卫生服务补助资金中医药标准制修订项目 (71) |
| 14 | 中医儿科临床诊疗指南·小儿泌尿道感染(修订 | 中医儿科杂志 | 2017 | 已更新 | 医院 | 诊疗 | 是 | 是 | 14 | 无 | 国家中医药管理局《2014年中医药部门公共卫生服务补助资金中医药标准制修订项目 (147) |
| 15 | 中医治未病实践指南·推拿干预小儿脾虚质(制订) | 中医儿科杂志 | 2017 | 否 | 医院 | 诊疗 | 是 | 是 | 14 | 无 | 国家中医药管理局《2014年中医药部门公共卫生服务补助资金中医药标准制修订项目 (44) |
| 16 | 中医儿科临床诊疗指南·神经性尿频(制订) | 中医儿科杂志 | 2017 | 否 | 医院 | 诊疗 | 是 | 是 | 22 | 无 | 国家中医药管理局《2014年中医药部门公共卫生服务补助资金中医药标准制修订项目 (66) |
| 17 | 中医儿科临床诊疗指南·流行性腮腺炎(修订） | 中医儿科杂志 | 2017 | 已更新 | 医院 | 诊疗 | 是 | 是 | 15 | 无 | 国家中医药管理局《2014年中医药部门公共卫生服务补助资金中医药标准制修订项目 (61) |
| 18 | .中医儿科临床诊疗指南·小儿汗证(制订) | 中医儿科杂志 | 2017 | 否 | 医院 | 诊疗 | 是 | 是 | 17 | 无 | 国家中医药管理局《2014年中医药部门公共卫生服务补助资金中医药标准制修订项目 (96) |
| 19 | Clinical practice guidelines of using acupuncture for low back pain | World Journal of Acupuncture Moxibustion (WJAM) | 2016 | 否 | 医院 | 诊疗 | 是 | 是 | 35 | 无 | X |
| 20 | .中医儿科临床诊疗指南·小儿急性肾小球肾炎(修订) | 中医儿科杂志 | 2016 | 已更新 | 医院 | 诊疗 | 是 | 是 | 16 | 无 | 国家中医药管理局《2014年中医药部门公共卫生服务补助资金中医药标准制修订项目 (62) |
| 21 | 中医儿科临床诊疗指南·小儿咳嗽变异性哮喘(制订) | 中医儿科杂志 | 2016 | 否 | 医院 | 诊疗 | 是 | 是 | 14 | 无 | 国家中医药管理局《2014年中医药部门公共卫生服务补助资金中医药标准制修订项目 (44) |
| 22 | .中医儿科临床诊疗指南·小儿免疫性血小板减少症(制订) | 中医儿科杂志 | 2016 | 否 | 医院 | 诊疗 | 是 | 是 | 25 | 无 | 国家中医药管理局《2014年中医药部门公共卫生服务补助资金中医药标准制修订项目 (67) |
| 23 | 中草药相关肝损伤临床诊疗指南 | 中国中药杂志 | 2016 | 否 | 医院 | 诊疗 | 是 | 是 | 37 | 未提及 | X |
| 24 | .中医儿科临床诊疗指南·性早熟(修订) | 中医儿科杂志 | 2016 | 已更新 | 医院 | 诊疗 | 是 | 是 | 17 | 无 | 国家中医药管理局《2014年中医药部门公共卫生服务补助资金中医药标准制修订项目 (110) |
| 25 | 中医儿科临床诊疗指南·小儿鼻鼽 | 中国中医药杂志 | 2016 | 否 | 医院 | 诊疗 | 是 | 是 | 26 | 无 | 国家中医药管理局《2014年中医药部门公共卫生服务补助资金中医药标准制修订项目 |
| 26 | .中医儿科临床诊疗指南·精神发育迟滞(制订) | 中医儿科杂志 | 2016 | 否 | 医院 | 诊疗 | 是 | 是 | 13 | 无 | 国家中医药管理局《2014年中医药部门公共卫生服务补助资金中医药标准制修订项目 (59) |
| 27 | 中医儿科临床诊疗指南·水痘(修订) | 中医儿科杂志 | 2016 | 已更新 | 医院 | 诊疗 | 是 | 是 | 20 | 无 | 国家中医药管理局《2014年中医药部门公共卫生服务补助资金中医药标准制修订项目 (59) |
| 28 | 蛇串疮中医诊疗指南 ( 2014 年修订版) | 中医杂志 | 2015 | 未提及 | 学会 | 诊疗 | 是 | 是 | 83 | 未提及 | 国家中医药管理局中医药标准化项目(ZYYS-2013PJ10) |
| 29 | 糖尿病足溃疡中医循证临床实践指南 | 中国中西医结合外科杂志 | 2015 | 提及 | 学会 | 诊疗 | 是 | 是 | 19 | 未提及 | 国家十一五科技支撑项目（2008BAI53B01） |
| 30 | 下肢慢性溃疡中医循证临床实践指南 | 中国中西医结合外科杂志 | 2015 | 提及 | 学会 | 诊疗 | 是 | 是 | 15 | 未提及 | 国家十一五科技支撑项目（2008BAI53B01） |
| 31 | 登革热诊疗指南_2014年第2版 | 传染病信息 | 2014 | 未提及 | 协会 | 诊疗 | 未提及 | 未提及 | X | 未提及 | X |
| 32 | 慢性肺原性心脏病中医诊疗指南_2014版_李建生 | 中医杂志 | 2014 | 未提及 | 协会 | 诊疗 | 是 | 未提及 | 25 | 未提及 | 国家中医临床研究基地业务建设科研专项 |
| 33 | 寻常型银屑病_白疕_中医药循证临床实践指南_2013版 | 中医杂志 | 2014 | 未提及 | 学会 | 综合 | 是 | 是 | 81 | 无 | 北京市科技计划资助项目 ( D09050703550901) |
| 34 | 慢性阻塞性肺疾病中医诊疗指南(2011版) | 中医杂志 | 2012 | 已更新 | 学会 | 诊疗 | 是 | 未提及 | 29 | 未提及 | 国家“十一五”科技支撑计划资助项目（2006BAI04A13）；国家自然科学基金资助项目（30772797） |
| 35 | 中国成人失眠诊断与治疗指南 | 中华神经科杂志 | 2012 | 未提及 | 学会 | 诊疗 | 未提及 | 未提及 | 67 | 未提及 | X |
| 36 | 中国卒中康复治疗指南简化版 | 中华神经科杂志 | 2012 | 未提及 | 学会 | 其他 | 未提及 | 未提及 | 65 | 未提及 | 国家“十一五”科技支撑计划“脑血管病规范化康复方案的研究” |
| 37 | 复发性阿弗他溃疡诊疗指南(试行) | 中华口腔医学杂志 | 2012 | 未提及 | 学会 | 诊疗 | 未提及 | 未提及 | 6 | 未提及 | X |
| 38 | 维生素D缺乏性佝偻病中医诊疗指南 | 中医儿科杂志 | 2012 | 将更新 | 医院 | 诊疗 | 是 | 是 | 10 | 无 | 国家中医药管理局中医药标准化项目[ZYYS-2009(0004)-34] |
| 39 | 中医药治疗手足口病临床技术指南 | X | 2012 | 已更新 | 卫生部 | 治疗 | 未提及 | 未提及 | X | 未提及 | X |
| 40 | 慢性乙型肝炎中医临床实践指南 | 专著 | 2011 | 已更新 | 中国中医科学院 | 诊疗 | 未提及 | 是 | 91 | 未提及 | WHO西太区资助项目 |
| 41 | 艾滋病中医临床实践指南 | 专著 | 2011 | 已更新 | 中国中医科学院 | 诊疗 | 未提及 | 是 | 29 | 未提及 | WHO西太区资助项目 |
| 42 | 原发性支气管肺癌中医临床实践指南 | 专著 | 2011 | 未提及 | 中国中医科学院 | 诊疗 | 未提及 | 是 | 54 | 未提及 | WHO西太区资助项目 |
| 43 | 再生障碍性贫血中医临床实践指南 | 专著 | 2011 | 已更新 | 中国中医科学院 | 诊疗 | 未提及 | 是 | 121 | 未提及 | WHO西太区资助项目 |
| 44 | 2型糖尿病中医临床实践指南 | 专著 | 2011 | 已更新 | 中国中医科学院 | 诊疗 | 未提及 | 是 | 89 | 未提及 | WHO西太区资助项目 |
| 45 | 单纯性肥胖病中医临床实践指南 | 专著 | 2011 | 已更新 | 中国中医科学院 | 诊疗 | 未提及 | 未提及 | 99 | 未提及 | WHO西太区资助项目 |
| 46 | 抑郁症中医临床实践指南 | 专著 | 2011 | 已更新 | 中国中医科学院 | 诊疗 | 未提及 | 是 | 66 | 未提及 | WHO西太区资助项目 |
| 47 | 失眠症中医临床实践指南 | 专著 | 2011 | 已更新 | 中国中医科学院 | 诊疗 | 未提及 | 是 | 10 | 未提及 | WHO西太区资助项目 |
| 48 | 血管性痴呆中医临床实践指南 | 专著 | 2011 | 已更新 | 中国中医科学院 | 诊疗 | 未提及 | 是 | 34 | 未提及 | WHO西太区资助项目 |
| 49 | 偏头痛中医临床实践指南 | 专著 | 2011 | 未提及 | 中国中医科学院 | 诊疗 | 未提及 | 是 | 52 | 未提及 | WHO西太区资助项目 |
| 50 | 高血压病中医临床实践指南 | 专著 | 2011 | 未提及 | 中国中医科学院 | 诊疗 | 是 | 是 | 72 | 未提及 | WHO西太区资助项目 |
| 51 | 慢性稳定性心绞痛中医临床实践指南 | 专著 | 2011 | 已更新 | 中国中医科学院 | 诊疗 | 是 | 是 | 195 | 未提及 | WHO西太区资助项目 |
| 52 | 脑梗死中医临床实践指南 | 专著 | 2011 | 已更新 | 中国中医科学院 | 诊疗 | 未提及 | 未提及 | 86 | 未提及 | WHO西太区资助项目 |
| 53 | 感冒中医临床实践指南 | 专著 | 2011 | 已更新 | 中国中医科学院 | 诊疗 | 未提及 | 未提及 | 65 | 未提及 | WHO西太区资助项目 |
| 54 | 2009甲型H1N1流感中医临床实践指南 | 专著 | 2011 | 已更新 | 中国中医科学院 | 诊疗 | 未提及 | 是 | 52 | 未提及 | WHO西太区资助项目 |
| 55 | 慢性胃炎中医临床实践指南 | 专著 | 2011 | 已更新 | 中国中医科学院 | 诊疗 | 未提及 | 是 | 125 | 未提及 | WHO西太区资助项目 |
| 56 | 类风湿关节炎中医临床实践指南 | 专著 | 2011 | 已更新 | 中国中医科学院 | 诊疗 | 未提及 | 是 | 62 | 未提及 | WHO西太区资助项目 |
| 57 | 原发性骨质疏松症中医临床实践指南 | 专著 | 2011 | 已更新 | 中国中医科学院 | 诊疗 | 未提及 | 是 | 108 | 未提及 | WHO西太区资助项目 |
| 58 | IgA肾病中医临床实践指南 | 专著 | 2011 | 已更新 | 中国中医科学院 | 诊疗 | 未提及 | 是 | 53 | 未提及 | WHO西太区资助项目 |
| 59 | 慢性阻塞性肺疾病中医临床实践指南 | 专著 | 2011 | 已更新 | 中国中医科学院 | 诊疗 | 未提及 | 是 | 109 | 未提及 | WHO西太区资助项目 |
| 60 | 年龄相关性黄斑变性（湿性）中医临床实践指南 | 专著 | 2011 | 已更新 | 中国中医科学院 | 诊疗 | 未提及 | 是 | 110 | 未提及 | WHO西太区资助项目 |
| 61 | 特应性皮炎中医临床实践指南 | 专著 | 2011 | 已更新 | 中国中医科学院 | 诊疗 | 未提及 | 是 | 97 | 未提及 | WHO西太区资助项目 |
| 62 | 寻常型银屑病中医临床实践指南 | 专著 | 2011 | 已更新 | 中国中医科学院 | 诊疗 | 未提及 | 是 | 102 | 未提及 | WHO西太区资助项目 |
| 63 | 神经根型颈椎病中医临床实践指南 | 专著 | 2011 | 已更新 | 中国中医科学院 | 诊疗 | 未提及 | 是 | 51 | 未提及 | WHO西太区资助项目 |
| 64 | 慢性前列腺炎中医临床实践指南 | 专著 | 2011 | 已更新 | 中国中医科学院 | 诊疗 | 未提及 | 是 | 39 | 未提及 | WHO西太区资助项目 |
| 65 | 慢性盆腔炎中医临床实践指南 | 专著 | 2011 | 已更新 | 中国中医科学院 | 诊疗 | 未提及 | 是 | 53 | 未提及 | WHO西太区资助项目 |
| 66 | 小儿肺炎中医临床实践指南 | 专著 | 2011 | 已更新 | 中国中医科学院 | 诊疗 | 未提及 | 是 | 48 | 未提及 | WHO西太区资助项目 |
| 67 | 桡骨远端骨折中医临床实践指南 | 专著 | 2011 | 已更新 | 中国中医科学院 | 诊疗 | 未提及 | 是 | 125 | 未提及 | WHO西太区资助项目 |
| 68 | 社区获得性肺炎中医诊疗指南_2011版_ | 中医杂志 | 2011 | 将更新 | 学会 | 诊疗 | 是 | 未提及 | 28 | 未提及 | 国家重点基础研究发展计划资助项目,河南省高校新世纪优秀人才支持计划资助项目 |
| 69 | 水痘中医诊疗指南 | 中医儿科杂志 | 2011 | 将更新 | 小组 | 诊疗 | 是 | 是 | 12 | 无 | 国家中医药管理局“中医儿科常见病诊疗指南" |
| 70 | 小儿病毒性肺炎中医诊疗指南 | 南京中医药大学学报 | 2011 | 将更新 | 学会 | 诊疗 | 是 | 是 | 21 | 无 | 国家科技部“中药新药临床评价研究技术平台(南京)建设”(2008ZX09312—004) |
| 71 | 小儿急性上呼吸道病毒感染中医诊疗指南 | 南京中医药大学学报 | 2011 | 将更新 | 学会 | 诊疗 | 是 | 是 | 15 | 无 | 国家科技部“中药新药临床评价研究技术平台(南京)建设”(2008ZX09312—005) |
| 72 | 便秘诊疗指南 | 中国中医药现代远程教育 | 2011 | 未提及 | 学会 | 诊疗 | 未提及 | 未提及 | X | 未提及 | 国家中医药管理局中医药标准化专题项目 |
| 73 | 喘病诊疗指南 | 中国中医药现代远程教育 | 2011 | 未提及 | 学会 | 诊疗 | 未提及 | 未提及 | X | 未提及 | 国家中医药管理局中医药标准化专题项目 |
| 74 | 多发性肌炎诊疗指南 | 中国中医药现代远程教育 | 2011 | 未提及 | 学会 | 诊疗 | 未提及 | 未提及 | X | 未提及 | 国家中医药管理局中医药标准化专题项目 |
| 75 | 肺胀诊疗指南 | 中国中医药现代远程教育 | 2011 | 未提及 | 学会 | 诊疗 | 未提及 | 未提及 | X | 未提及 | 国家中医药管理局中医药标准化专题项目 |
| 76 | 功能性便秘诊疗指南 | 中国中医药现代远程教育 | 2011 | 未提及 | 学会 | 诊疗 | 未提及 | 未提及 | X | 未提及 | 国家中医药管理局中医药标准化专题项目 |
| 77 | 鼓胀诊疗指南 | 中国中医药现代远程教育 | 2011 | 未提及 | 学会 | 诊疗 | 未提及 | 未提及 | X | 未提及 | 国家中医药管理局中医药标准化专题项目 |
| 78 | 过敏性紫癜中医诊疗指南 | 中医儿科杂志 | 2011 | 将更新 | 学会 | 诊疗 | 是 | 是 | 18 | 无 | 国家中医药管理局“ 中医儿科常见病诊疗指南”资助项目 |
| 79 | 黄疸诊疗指南 | 中国中医药现代远程教育 | 2011 | 未提及 | 学会 | 诊疗 | 未提及 | 未提及 | X | 未提及 | 国家中医药管理局中医药标准化专题项目 |
| 80 | 急性气管_支气管炎诊疗指南 | 中国中医药现代远程教育 | 2011 | 未提及 | 学会 | 诊疗 | 未提及 | 未提及 | X | 未提及 | 国家中医药管理局中医药标准化专题项目 |
| 81 | 急性肾小球肾炎诊疗指南 | 中国中医药现代远程教育 | 2011 | 未提及 | 学会 | 诊疗 | 未提及 | 未提及 | X | 未提及 | 国家中医药管理局中医药标准化专题项目 |
| 82 | 溃疡性结肠炎诊疗指南 | 中国中医药现代远程教育 | 2011 | 未提及 | 学会 | 诊疗 | 未提及 | 未提及 | X | 未提及 | 国家中医药管理局中医药标准化专题项目 |
| 83 | 溃疡性结肠炎中西医结合诊疗指南（草案） | 中国中西医结合消化杂志 | 2011 | 未提及 | 学会 | 诊疗 | 未提及 | 未提及 | 21 | 未提及 | X |
| 84 | 类风湿性关节炎诊疗指南 | 中国中医药现代远程教育 | 2011 | 未提及 | 学会 | 诊疗 | 未提及 | 未提及 | X | 未提及 | 国家中医药管理局中医药标准化专题项目 |
| 85 | 慢性肾衰竭诊疗指南 | 中国中医药现代远程教育 | 2011 | 未提及 | 学会 | 诊疗 | 未提及 | 未提及 | X | 未提及 | 国家中医药管理局中医药标准化专题项目 |
| 86 | 慢性肾小球肾炎诊疗指南 | 中国中医药现代远程教育 | 2011 | 未提及 | 学会 | 诊疗 | 未提及 | 未提及 | X | 未提及 | 国家中医药管理局中医药标准化专题项目 |
| 87 | 慢性胃炎诊疗指南 | 中国中医药现代远程教育 | 2011 | 未提及 | 学会 | 诊疗 | 未提及 | 未提及 | X | 未提及 | 国家中医药管理局中医药标准化专题项目 |
| 88 | 慢性阻塞性肺疾病诊疗指南 | 中国中医药现代远程教育 | 2011 | 未提及 | 学会 | 诊疗 | 未提及 | 未提及 | X | 未提及 | 国家中医药管理局中医药标准化专题项目 |
| 89 | 呕吐诊疗指南 | 中国中医药现代远程教育 | 2011 | 未提及 | 学会 | 诊疗 | 未提及 | 未提及 | X | 未提及 | 国家中医药管理局中医药标准化专题项目 |
| 90 | 湿疹诊疗指南(2011年) | 中华皮肤科杂志 | 2011 | 未提及 | 学会 | 诊疗 | 未提及 | 未提及 | 8 | 未提及 | X |
| 91 | 糖尿病肾病中医防治指南 | 中国中医药现代远程教育 | 2011 | 未提及 | 学会 | 防治 | 未提及 | 未提及 | X | 未提及 | 国家中医药管理局中医药标准化专题项目 |
| 92 | 糖尿病视网膜病变中医防治指南 | 中国中医药现代远程教育 | 2011 | 未提及 | 学会 | 防治 | 未提及 | 未提及 | X | 未提及 | 国家中医药管理局中医药标准化专题项目 |
| 93 | 糖尿病中医防治指南 | 中国中医药现代远程教育 | 2011 | 未提及 | 学会 | 防治 | 未提及 | 未提及 | X | 未提及 | 国家中医药管理局中医药标准化专题项目 |
| 94 | 糖尿病中医防治指南糖尿病合并脑血管病 | 中国中医药现代远程教育 | 2011 | 未提及 | 学会 | 防治 | 未提及 | 未提及 | X | 未提及 | 国家中医药管理局中医药标准化专题项目 |
| 95 | 糖尿病中医防治指南糖尿病足 | 中国中医药现代远程教育 | 2011 | 未提及 | 学会 | 防治 | 未提及 | 未提及 | X | 未提及 | 国家中医药管理局中医药标准化专题项目 |
| 96 | 胃脘痛诊疗指南 | 中国中医药现代远程教育 | 2011 | 未提及 | 学会 | 诊疗 | 未提及 | 未提及 | X | 未提及 | 国家中医药管理局中医药标准化专题项目 |
| 97 | 胃下垂诊疗指南 | 中国中医药现代远程教育 | 2011 | 未提及 | 学会 | 诊疗 | 未提及 | 未提及 | X | 未提及 | 国家中医药管理局中医药标准化专题项目 |
| 98 | 系统性红斑狼疮诊疗指南 | 中国中医药现代远程教育 | 2011 | 未提及 | 学会 | 诊疗 | 未提及 | 未提及 | X | 未提及 | 国家中医药管理局中医药标准化专题项目 |
| 99 | 纤维肌痛综合征诊断和治疗指南 | 中华风湿病学杂志 | 2011 | 未提及 | 学会 | 诊疗 | 未提及 | 未提及 | X | 未提及 | X |
| 100 | 哮病诊疗指南 | 中国中医药现代远程教育 | 2011 | 未提及 | 学会 | 诊疗 | 未提及 | 未提及 | X | 未提及 | 国家中医药管理局中医药标准化专题项目 |
| 101 | 胁痛诊疗指南 | 中国中医药现代远程教育 | 2011 | 未提及 | 学会 | 诊疗 | 未提及 | 未提及 | X | 未提及 | 国家中医药管理局中医药标准化专题项目 |
| 102 | 血管性认知障碍诊治指南 | 中华神经科杂志 | 2011 | 未提及 | 学会 | 诊疗 | 未提及 | 未提及 | 68 | 未提及 | 十一五国家科技支撑计划基金资助项目(2006BAI02B01) |
| 103 | 中医内科常见病诊疗指南(西医疾病部分)病毒性心肌炎 | 中国中医药现代远程教育 | 2011 | 未提及 | 学会 | 诊疗 | 未提及 | 未提及 | X | 未提及 | 国家中医药管理局中医药标准化专题项目 |
| 104 | 中医内科常见病诊疗指南(西医疾病部分)冠心病心绞痛 | 中国中医药现代远程教育 | 2011 | 未提及 | 学会 | 诊疗 | 未提及 | 未提及 | X | 未提及 | 国家中医药管理局中医药标准化专题项目 |
| 105 | 中医内科常见病诊疗指南(西医疾病部分)室性早搏 | 中国中医药现代远程教育 | 2011 | 未提及 | 学会 | 诊疗 | 未提及 | 未提及 | X | 未提及 | 国家中医药管理局中医药标准化专题项目 |
| 106 | 中医内科常见病诊疗指南(西医疾病部分)心力衰竭 | 中国中医药现代远程教育 | 2011 | 未提及 | 学会 | 诊疗 | 未提及 | 未提及 | X | 未提及 | 国家中医药管理局中医药标准化专题项目 |
| 107 | 中国急性缺血性脑卒中诊治指南2010 | 中华神经科杂志 | 2010 | 已更新 | 学会 | 诊疗 | 是 | 未提及 | 58 | 未提及 | X |
| 108 | 儿童甲型H1N1流感中医药防治指南 | 中华中医药杂志 | 2010 | 未提及 | 学会 | 防治 | 未提及 | 未提及 | 2 | 未提及 | X |
| 109 | 甲型H1N1流感诊疗方案(2010年版) | 中华中医药杂志 | 2010 | 已更新 | 学会 | 防治 | 未提及 | 未提及 | 2 | 无 | 国家自然科学基金资助项目（No.30772766 ）；江苏省自然科学基金资助项目（No.BK2007239） |
| 110 | 激素依赖性皮炎诊治指南 | 临床皮肤科杂志 | 2009 | 未提及 | 协会 | 诊疗 | 未提及 | 未提及 | 8 | 未提及 | X |
| 111 | 生殖器疱疹中西医结合诊疗指南 | 中国中西医结合皮肤性病学杂志 | 2009 | 将更新 | 学会 | 诊疗 | 未提及 | 未提及 | X | 未提及 | X |
| 112 | 中国银屑病治疗指南2008版 | 中华皮肤科杂志 | 2009 | 未提及 | 学会 | 治疗 | 未提及 | 未提及 | X | 未提及 | X |
| 113 | 甲型H1N1流感密切接触者中相关人员预防性用药指南2009年试行版 | 中华医学杂志 | 2009 | 未提及 | 卫生部 | 预防 | 未提及 | 未提及 | X | 未提及 | X |
| 114 | 慢性鼻－鼻窦炎诊断和治疗指南（2008） | 中华耳鼻咽喉头颈外科杂志 | 2009 | 未提及 | 综合 | 诊疗 | 未提及 | 未提及 | 8 | 未提及 | X |
| 115 | 小儿感冒中医诊疗指南 | 中医儿科杂志 | 2009 | 未提及 | 学会 | 诊疗 | 未提及 | 未提及 | X | 未提及 | X |
| 116 | 中国0至5岁儿童病因不明的急性发热诊断处理指南(标准版) | 中国循证儿科杂志 | 2008 | 未提及 | 杂志 | 诊疗 | 是 | 未提及 | 149 | 是 | 未接受外来资助 |
| 117 | 中国痤疮治疗指南（讨论稿） | 临床皮肤科杂志 | 2008 | 将更新 | 协会 | 治疗 | 未提及 | 未提及 | X | 未提及 | X |
| 118 | 白癜风维吾尔医诊疗指南 | 中国民族医药杂志 | 2008 | 未提及 | 医院 | 诊疗 | 未提及 | 未提及 | X | 是 | 国家中医药管理局“维吾尔医临床技术操作规范与病证诊疗指南”项目, ZYYS-2007-0026 |
| 119 | 溃疡性结肠炎维吾尔医诊疗指南 | 中国民族医药杂志 | 2008 | 未提及 | 医院 | 诊疗 | 未提及 | 未提及 | X | 未提及 | X |
| 120 | 流行性腮腺炎中医诊疗指南 | 中医儿科杂志 | 2008 | 未提及 | 学会 | 诊疗 | 未提及 | 未提及 | X | 未提及 | X |
| 121 | 慢性稳定性心绞痛维吾尔医诊疗指南 | 中国民族医药杂志 | 2008 | 未提及 | 医院 | 诊疗 | 未提及 | 未提及 | X | 无 | 国家中医药管理局维吾尔医临床技术操作规范与病证诊疗指南项目 |
| 122 | 膝骨关节炎维吾尔医诊疗指南 | 中国民族医药杂志 | 2008 | 未提及 | 医院 | 诊疗 | 未提及 | 未提及 | X | 无 | 国家中医药管理局维吾尔医临床技术操作规范与病证诊疗指南项目 |
| 123 | 小儿反复呼吸道感染中医诊疗指南 | 中医儿科杂志 | 2008 | 未提及 | 学会 | 诊疗 | 未提及 | 未提及 | X | 未提及 | X |
| 124 | 小儿肺炎喘嗽中医诊疗指南 | 中医儿科杂志 | 2008 | 未提及 | 学会 | 诊疗 | 未提及 | 未提及 | X | 未提及 | X |
| 125 | 小儿哮喘中医诊疗指南 | 中医儿科杂志 | 2008 | 未提及 | 学会 | 诊疗 | 未提及 | 未提及 | X | 未提及 | X |
| 126 | 小儿泄泻中医诊疗指南 | 中医儿科杂志 | 2008 | 未提及 | 学会 | 诊疗 | 未提及 | 未提及 | X | 未提及 | X |
| 127 | 小儿支气管炎中医诊疗指南 | 中医儿科杂志 | 2008 | 未提及 | 学会 | 诊疗 | 未提及 | 未提及 | X | 未提及 | X |
| 128 | 银屑病维吾尔医诊疗指南 | 中国民族医药杂志 | 2008 | 未提及 | 医院 | 诊疗 | 未提及 | 未提及 | X | 无 | 国家中医药管理局“维吾尔医临床技术操作规范与病证诊疗指南”项目 |
| 129 | 子宫颈糜烂的维吾尔医护理技术操作规范 | 中国民族医药杂志 | 2008 | 未提及 | 医院 | 技术 | 未提及 | 未提及 | X | 无 | 国家中医药管理局“维吾尔医临床技术操作规范与病证诊疗指南”项目，ZY'YS20070026． |
| 130 | 子宫颈糜烂维吾尔医诊疗指南 | 中国民族医药杂志 | 2008 | 未提及 | 医院 | 诊疗 | 未提及 | 未提及 | X | 无 | 国家中医药管理局维吾尔医临床技术操作规范与病证诊疗指南项目 |
| 131 | 子宫平滑肌瘤的维吾尔医护理技术操作规范 | 中国民族医药杂志 | 2008 | 未提及 | 医院 | 技术 | 未提及 | 未提及 | X | 无 | 国家中医药管理局“维吾尔医临床技术操作规范与病证诊疗指南”项目 |
| 132 | 子宫平滑肌瘤维吾尔医诊疗指南 | 中国民族医药杂志 | 2008 | 未提及 | 医院 | 诊疗 | 未提及 | 未提及 | X | 无 | 国家中医药管理局“维吾尔医临床技术操作规范与病证诊疗指南”项目 |
| 133 | 慢性前列腺炎中西医结合诊疗指南试行版 | 中国中西医结合杂志 | 2007 | 未提及 | 学会 | 诊疗 | 未提及 | 未提及 | 24 | 未提及 | X |
| 134 | 慢性阻塞性肺疾病诊治指南2007年修订版 | 中华结核和呼吸杂志 | 2007 | 已更新 | 学会 | 诊疗 | 未提及 | 未提及 | 7 | 未提及 | X |
| 135 | 脾损伤脾保留手术操作建议指南 | 中国实用外科杂志 | 2007 | 未提及 | 学会 | 技术 | 未提及 | 未提及 | X | 未提及 | X |
| 136 | 心肺复苏与中西医结合急救指南(草案) | 中国中医急症 | 2007 | 未提及 | 学会 | 诊疗 | 未提及 | 未提及 | 14 | 未提及 | X |
| 137 | 中国肥胖病外科治疗指南(2007) | 中华实用外科杂志 | 2007 | 未提及 | 学会 | 治疗 | 未提及 | 未提及 | 20 | 未提及 | X |
| 138 | 肝纤维化中西医结合诊疗指南 | 中华肝脏病杂志 | 2006 | 未提及 | 学会 | 诊疗 | 未提及 | 未提及 | 34 | 未提及 | X |
| 139 | 痔临床诊治指南(2006版) | 中华胃肠外科杂志 | 2006 | 已更新 | 学会 | 诊疗 | 未提及 | 未提及 | X | 未提及 | X |
| 140 | 流行性感冒临床诊断和治疗指南2004年修订稿 | 中华结核和呼吸杂志 | 2005 | 未提及 | 学会 | 诊疗 | 未提及 | 未提及 | 10 | 未提及 | X |
| 141 | 传染性非典型肺炎SARS诊疗方案 | 中华医学杂志 | 2003 | 未提及 | 学会 | 诊疗 | 未提及 | 未提及 | 10 | 未提及 | X |
| 142 | 急性胰腺炎诊治指南讨论稿 | 中华胰腺病杂志 | 2003 | 未提及 | 学会 | 诊疗 | 未提及 | 未提及 | X | 未提及 | X |

X 表示未报告
